# Effects of different running intensities on the micro-level failure strain of rat femoral cortical bone structures: a finite element investigation

**DOI:** 10.1186/s12938-023-01151-6

**Published:** 2023-09-12

**Authors:** Ruoxun Fan, Jie Liu, Zhengbin Jia

**Affiliations:** 1 Department of Traffic Engineering, Yangzhou Polytechnic Institute, Yangzhou, 225127 People’s Republic of China; 2 Department of Aerospace Engineering, Jilin Institute of Chemical Technology, Jilin, 132022 People’s Republic of China; 3 Department of Engineering Mechanics, Jilin University, Changchun, 130022 People’s Republic of China

**Keywords:** Cortical bone, Three-point bending load, Micro-level failure strain, Running intensity, Back-calculation

## Abstract

**Background:**

Running with the appropriate intensity may produce a positive influence on the mechanical properties of cortical bone structure. However, few studies have discussed the effects of different running intensities on the mechanical properties at different levels, especially at the micro-level, because the micromechanical parameters are difficult to measure experimentally.

**Methods:**

An approach that combines finite element analysis and experimental data was proposed to predict a micromechanical parameter in the rat femoral cortical bone structure, namely, the micro-level failure strain. Based on the previous three-point bending experimental information, fracture simulations were performed on the femur finite element models to predict their failure process under the same bending load, and the micro-level failure strains in tension and compression of these models were back-calculated by fitting the experimental load–displacement curves. Then, the effects of different running intensities on the micro-level failure strain of rat femoral cortical bone structure were investigated.

**Results:**

The micro-level failure strains of the cortical bone structures expressed statistical variations under different running intensities, which indicated that different mechanical stimuli of running had significant influences on the micromechanical properties. The greatest failure strain occurred in the cortical bone structure under low-intensity running, and the lowest failure strain occurred in the structure under high-intensity running.

**Conclusions:**

Moderate and low-intensity running were effective in enhancing the micromechanical properties, whereas high-intensity running led to the weakening of the micromechanical properties of cortical bone. Based on these, the changing trends in the micromechanical properties were exhibited, and the effects of different running intensities on the fracture performance of rat cortical bone structures could be discussed in combination with the known mechanical parameters at the macro- and nano-levels, which provided the theoretical basis for reducing fracture incidence through running exercise.

## Background

Cortical bone bears most of the external loads, and once damage occurs in the cortical bone structure, cracks will propagate under different loads and eventually lead to fracture [1, 2]. The inevitable degeneration in the mechanical properties of cortical bone with age will further increase the incidence of fracture [3]. Therefore, exploring an appropriate method to improve the mechanical properties to reduce fracture incidence is important in the research field of biomechanics.

Running exercise is recognized as an effective way to improve bone mechanical properties, and running with the appropriate intensity could increase bone density and strength [4, 5]. Several experiments considered that the fracture load, cortical bone thickness, and tissue-level elastic modulus in the rat femoral and tibial cortical bone structures significantly increased under treadmill running at low or moderate intensity [6–8]. However, different viewpoints exist in the studies on the effects of high-intensity running on the mechanical properties of cortical bone. Several studies found that the rat femoral cortical bone under high-intensity running was inferior to that of the sedentary group in terms of apparent mechanical properties and microstructural morphological parameters [9, 10]. Moreover, a report suggested that high-intensity running increased cortical bone density and thickness but not ultimate strength [11]. Therefore, the effects of different running intensities on the mechanical properties of cortical bone structure remain to be further investigated.

Furthermore, comparisons of several experimental results found different changing trends in the mechanical parameters at the macro-, micro-, and nano-levels even under the same running intensity. For example, the mechanical stimuli of running did not change the tissue-level elastic modulus and hardness but improved the microstructural morphology [12]. The main reason for this phenomenon is that cortical bone is hierarchical with its overall mechanical response being influenced by the interplay of its structure and material composition at different levels [13, 14]. Thus, mastering the changes in mechanical parameters at different levels is necessary to investigate the effects of different running intensities on the mechanical properties of cortical bone. Current experiments can obtain the macro- and nano-levels mechanical parameters but have difficulties in measuring the micro-level mechanical parameters in cortical bone structure [15, 16]. Most studies only reported the changes in the microstructural morphology parameters under different loading environments [6, 12]. The effects of micromechanical parameters on cortical bone fracture performance are significant, especially for micro-level failure strain in the osteon [17]. The softening and fracture time of bone structure are partly determined by the micro-level failure strain, and the processes of bone resorption and formation are also influenced by the micro-level failure strain [18, 19]. These illustrate that micromechanical properties are of great importance in the mechanics and physiology of cortical bone structure. Therefore, accurate acquisition of micromechanical parameters is a prerequisite for mastering fracture performance and predicting fracture risk in cortical bone structure.

The purpose of this paper was to propose an approach that combines finite element (FE) analysis and experimental data to predict the micro-level failure strain for the rat femoral cortical bone structures. FE analyses were performed on the rat femoral cortical bone structures to simulate their failure processes under three-point bending load. Different failure strains were repeatedly assigned to the FE models during simulation, and the suitable values could be back-calculated by fitting the load–displacement curves between the experiments and simulations. Then, the effects of different running intensities on the micro-level failure strain in the cortical bone structures were investigated, which provided the theoretical basis for reducing fracture incidence through running exercise.

## Results

### Prediction results of micro-level failure strain

**Fig. 1:**
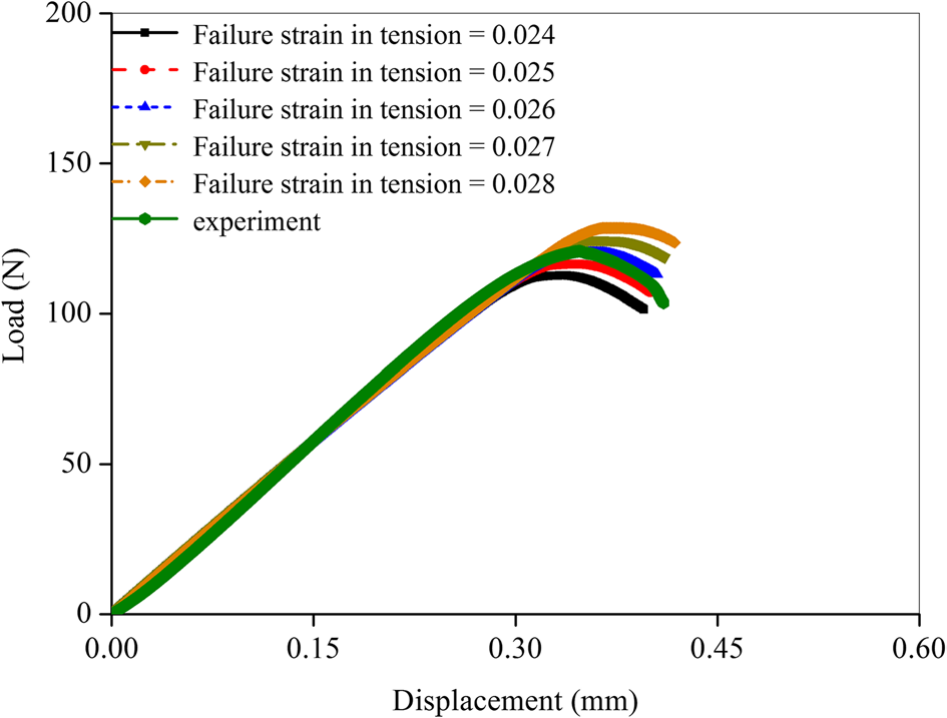
Comparison of the prediction precision with different tensile failure strains

Fracture simulations were performed on the forty-eight femoral FE models to predict the tensile and compressive micro-level failure strains of cortical bone structures in the different rat groups. All the material input parameters, except the failure strain, have been measured in the previous experiments; therefore, the fitting accuracy in the load–displacement curves depended on the assignment of the failure strain. The adjustment interval of the tensile failure strain was set to 0.001 to ensure the simulation precision. That was, each simulation was conducted by adding 0.001 to the last tensile strain and then re-running the fracture simulation until the fit was successful. Figure 1 shows the comparison of the prediction precision with different tensile failure strains. A complete fracture occurred later as tensile failure strain increased, resulting in an increase in fracture load. The discrepancies in the load–displacement curves were not remarkable and the differences in the fracture parameters between every two curves were less than 5% when the adjustment interval of the tensile failure strain was set to 0.001. Therefore, this comparison indicated that setting the tensile strain adjustment interval to 0.001 was reasonable when performing fitting.

The fitting process was completed when the differences in the fracture parameters between the experiment and simulation were less than 5%, and the predicted tensile and compressive failure strains in the FE models were the micro-level failure strains of the cortical bone structure. The predicted micro-level failure strains in the different groups are shown in Table 1. Significant differences in micro-level failure strain were detected in the rat cortical bone structures under treadmill running with different intensities, which indicated that the different mechanical stimuli of running had a significant influence on the micromechanical properties. The greatest failure strain occurred in the cortical bone structure under low-intensity treadmill running, and the lowest failure strain occurred under high-intensity treadmill running.

**Table 1:** The predicted micro-level failure strains of cortical bone structures in different groups, mean ± SD

Rat group	Micro-level failure strain in tension	Micro-level failure strain in compression
SED	0.0275 ± 0.000313	0.0458 ± 0.000521
EX12	0.0289 ± 0.000391	0.0483 ± 0.000651
EX16	0.0284 ± 0.000317	0.0473 ± 0.000529
EX20	0.0251 ± 0.000319	0.0417 ± 0.000532

All the data were statistically different among the four groups (*p* < 0.05)

**Fig. 2:**
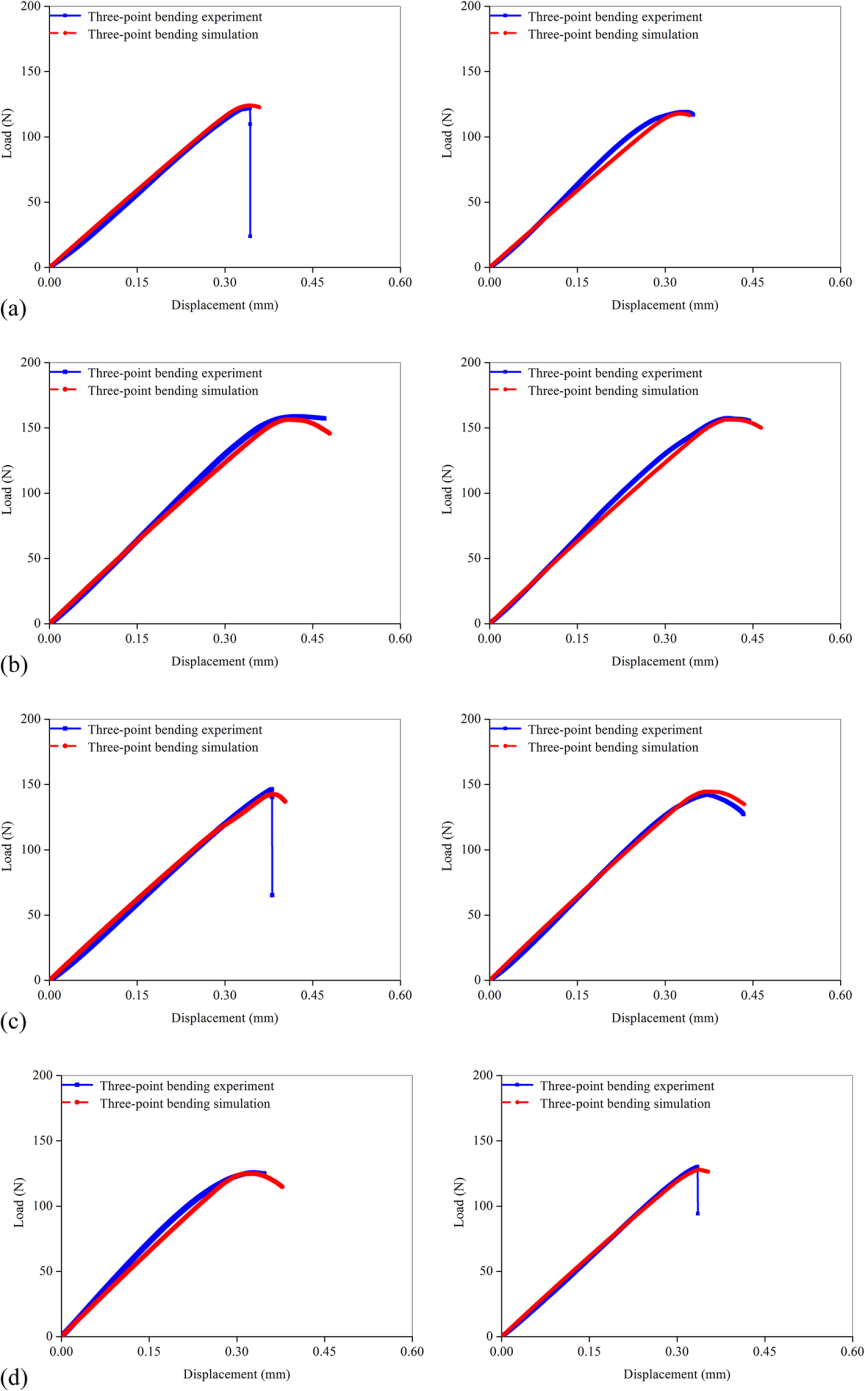
The load–displacement curves obtained from the experiment and simulation in each rat group. **a** SED group; **b** EX12 group; **c** EX16 group; **d** EX20 group

Moreover, the load–displacement curves of the two femur FE models and the corresponding cortical bone samples were selected from each group for observation, as shown in Fig. 2. Meanwhile, Table 2 shows the apparent stiffness and fracture load of the samples in the previous experiment. Because the input material parameters and the femur micro-CT images were both obtained from previous experiments, the established femur FE models should be similar to the experimental cortical bone samples. Thus, the simulated and experimental load–displacement curves could be well fitted, and the fracture parameters between the experiments and simulations were nearly the same. The comparison of the curves showed that quasi-brittle fracture occurred in the three-point bending experiment and simulation, which indicated that the structure experienced a long elastic stage and then entered the fracture stage. Thus, the micro-level failure strain could be back-calculated directly from fitting the load–displacement curves because the yield stage was not obvious.

**Table 2:** The fracture parameters of the femur samples measured in the previous experiment, mean ± SD

Rat group	Apparent stiffness (GPa)	Fracture load (N)
SED	6.85 ± 2.72	124.52 ± 30.50
EX12	7.39 ± 1.32	155.62 ± 20.77^a,b^
EX16	7.24 ± 1.95	142.21 ± 33.71
EX20	8.00 ± 2.76	122.49 ± 24.17

^a^Statistically different from SED (*p* < 0.05)

^b^Statistically different from EX20 (*p* < 0.05)

### The microarchitecture parameters in the rat femoral cortical bone

The microarchitecture parameters of the rat femoral cortical bone structures were calculated with CTAn, as shown in Table 3. Statistically greater Ct.BMD was detected in the EX12 group compared with the SED and EX16 groups (*p* < 0.05). Statistically greater Ct.Th was detected in the EX12 group compared with the SED and EX20 groups (*p* < 0.05). Significantly greater Ct.P was detected in the EX16 group compared with the SED and EX12 groups (*p* < 0.05).

**Table 3:** Microarchitecture parameters of the femoral cortical bone evaluated by micro-CT, mean ± SD

Rat group	Ct.BMD (g/cm^3^)	Ct.Th (mm)	Ct.P (%)
SED	1.67 ± 0.03	32.40 ± 3.32	2.51 ± 0.60
EX12	1.71 ± 0.03^a,c^	35.23 ± 2.97^a,d^	2.25 ± 0.45
EX16	1.67 ± 0.05	34.41 ± 4.48	3.17 ± 0.71^a,b^
EX20	1.68 ± 0.03	32.40 ± 2.50	2.67 ± 0.59

^a^Statistically different from SED (*p* < 0.05)

^b^Statistically different from EX12 (*p* < 0.05)

^c^Statistically different from EX16 (*p* < 0.05)

^d^Statistically different from EX20 (*p* < 0.05)

## Discussion

The cortical bone structure may express different changing magnitudes and trends in the mechanical properties at different levels even under the same load owing to its hierarchical structural characteristic [20]. Therefore, mastering the mechanical parameters at the macro- and micro-levels is necessary to investigate the effects of different loading conditions on the fracture performance of cortical bone structure. Micromechanical parameters are difficult to measure experimentally because of the limitations in the structural characteristics. Thus, this study aimed to propose an approach to predict a micro-level mechanical parameter in the cortical bone structure and observe the changes in this mechanical parameter under running with different intensities.

**Fig. 3:**
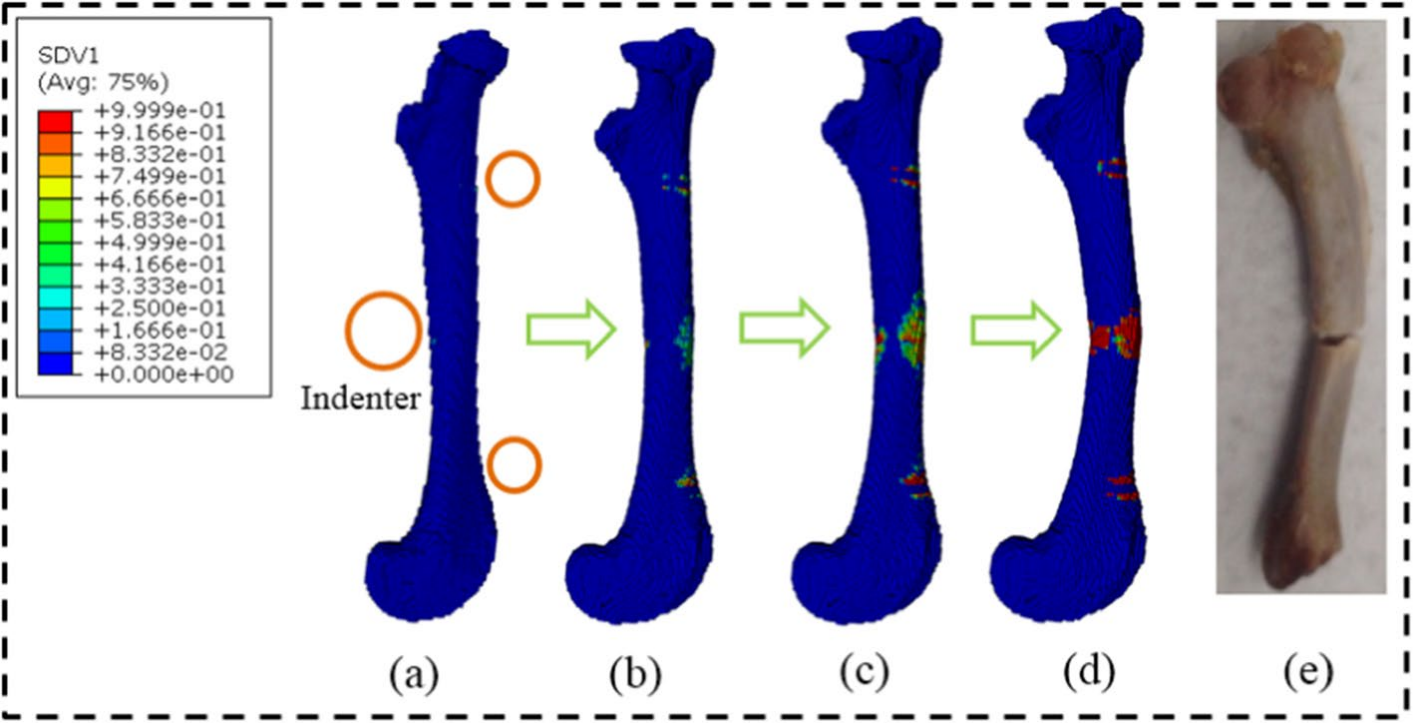
The failure process in the FE model under three-point bending load and the comparison of the fracture patterns between the present simulation and the previous experiment. The “SDV1” represents the damage variable *D*. **a** initial loading stage; **b** element damage stage; **c** element failure stage; **d** structure fracture stage; **e** experimental fracture pattern

One of the advantages of FE analysis is that the mechanical state in the FE model can be observed at any time [21]. Figure 3 exhibits the complete failure process in the femur FE model under three-point bending load. At the initial loading stage, the principal strain was high at the loading and constraint locations in the femur, and few green damaged elements first appeared near the upper indenter, as shown in Fig. 3a. Then, a large number of green damaged elements appeared at the lower middle of the femoral shaft with bending, which was opposed to the upper indenter, as shown in Fig. 3b. When the bending reached a certain degree, the red failed elements appeared at the lower side of the cortical bone structure and expanded longitudinally from the middle to both sides, forming a fan-shaped failure region, as shown in Fig. 3c. In addition, because the principle strain increased more quickly at both sides of the lower cylinders due to the support during bending, the elements near the lower cylinders occurred failure at the middle loading stage. However, no more new damaged elements occurred, which illustrated that the crack in the femur under bending load propagated in the middle position but not in the constraint location. When the red failed elements in the lower part of the cortical bone extended to both sides to a certain degree, they extended transversely and upward instead of longitudinally along the femur until the failed elements penetrated the central section of the femoral shaft, resulting in complete fracture, as shown in Fig. 3d. The comparison stated that the fracture pattern in the simulation was consistent with the previous experimental result, as shown in Fig. 3e [22]. Furthermore, the femur fracture under three-point bending load has been reported to be caused by the continuous propagation of the lower surface crack in transverse and longitudinal directions, which cuts off the central section of the femoral shaft [23]. These comparisons could verify the feasibility and accuracy of the simulation in this study.

The results showed that the micro-level failure strains of cortical bone structure in the four rat groups were statistically different from each other (*p* < 0.05), which indicated that different mechanical stimuli of running produced significant effects on the micromechanical properties of cortical bone structure. The EX12 group generated the greatest improvement in the micro-level failure strain, and the failure strain in the EX20 group was significantly lower than that in the SED group. The micro-level failure strain represents the failure threshold in the osteon, so its value could be used to describe the micromechanical properties of the structure and partly determine the apparent fracture process [24, 25]. Thus, this finding suggested that moderate and low-intensity running were effective in enhancing the micromechanical properties, whereas high-intensity running led to the weakening of the micromechanical properties of cortical bone structure.

A comprehensive discussion of the mechanical parameters at different levels is necessary to investigate the effects of different running intensities on the fracture performance of cortical bone owing to its hierarchical structural organization. Previous experiment showed that the fracture load and apparent stiffness increased in the EX12 and EX16 groups compared with the SED group, as shown in Table 2. Macro-level mechanical parameters are often determined by the combination of mechanical parameters at the micro- and nano-levels and microstructural morphology [26, 27]. Thus, the fracture load should be determined by a combination of microarchitecture parameters, micro-level failure strain, and tissue-level elastic modulus. The statistical results of the microarchitecture parameters in this study showed that the Ct.Th in the EX12 and EX16 groups was statistically greater than those in the SED and EX20 groups, and the EX16 group had a remarkably greater Ct.P compared with the EX12 group. Moreover, the tissue-level elastic modulus is significantly greater in the EX12 and EX16 groups than in the other two groups, as shown in Table 4. The differences in the predicted micro-level failure strains suggested that the micro- and nano-levels mechanical properties in the EX12 and EX16 groups were better than those in the other two groups, and their mechanical properties at the macro-level, including fracture load and apparent stiffness, were better than those of the other two groups. Therefore, running at moderate and low intensities could improve the mechanical properties of cortical bone structure.

**Table 4:** The tissue-level elastic modulus of the cortical bone measured by nanoindentation test, mean ± SD

Groups	Longitudinal elastic modulus (GPa)	Transverse elastic modulus (GPa)
SED	23.57 ± 3.85	19.69 ± 2.97
EX12	24.15 ± 3.85	21.56 ± 2.29^a,d^
EX16	25.04 ± 3.84	20.97 ± 1.83^a,d^
EX20	20.71 ± 3.03^a,b,c^	18.74 ± 1.67

^a^Statistically different from SED (*p* < 0.05)

^b^Statistically different from EX12 (*p* < 0.05)

^c^Statistically different from EX16 (*p* < 0.05)

^d^Statistically different from EX20 (*p* < 0.05)

The previous experiment also showed that the fracture load was the lowest in the EX20 group, but the apparent stiffness was greater compared with the other groups, as shown in Table 2. This phenomenon states that different macro-level mechanical parameters in one cortical bone structure may also express different variations. The reason may be that the macro-level mechanical parameter was determined by different micro- and nano-levels parameters. No substantial differences in the microarchitecture parameters are found between the SED and EX20 groups in Table 3. Although the longitudinal elastic modulus in the EX20 group was significantly lower than that in the SED group, the femur fracture under three-point bending load was mainly caused by transverse deformation, which depended on the transverse elastic modulus [28]. No remarkable differences in the transverse elastic modulus existed between the EX20 and SED groups, suggesting that the differences in fracture performance between the EX20 and SED groups may be determined by the micromechanical properties. The simulation results showed that the micro-level failure strain in the EX20 group was substantially lower than those in the other groups, which led to advanced fracture and low fracture load. However, its apparent stiffness was not low because of the better microstructure morphology [29]. Therefore, the fracture load in the EX20 group was lower than that in the SED group mainly because of the decrease in micro-level failure strain.

Several limitations existed during the simulations in this study. First, the loading condition was single, only the three-point bending load was considered. In reality, the femur may be subjected to compression, torsion, and impact loads. Although this study only simulated the cortical bone fracture under three-point bending load, the established numerical method may also be applicable to bone fractures caused by other types of loads. Second, the microarchitecture features in the fracture surface have a great influence on the fracture performance. Our previous experiment did not conduct scanning electron microscopy analysis on the cortical bone fracture surface; hence, the microarchitecture features of the fracture surface could not be observed. However, the reasons for the changes in the macro-level mechanical parameters and the relationships between microarchitecture and micro-level failure strain under different mechanical stimuli were discussed, which may compensate for the inability to investigate the effects of the fracture surface on the microarchitecture features.

## Conclusions

The failure process in the rat femur FE models under three-point bending load was simulated, and the micro-level failure strain was predicted by back-calculation with the experimental data. The micro-level failure strains of cortical bone structures in the four rat groups were statistically different from each other, which indicated that different mechanical stimuli of running produced significant effects on the micromechanical properties of cortical bone structure. The greatest micro-level failure strain occurred in the cortical bone structure under low-intensity running, and the lowest failure strain occurred in the structure under high-intensity running. This finding suggested that moderate and low-intensity running were effective in enhancing the micromechanical properties, whereas high-intensity running led to the weakening of the micromechanical properties of cortical bone structure.

## Materials and methods

### Description of the previous three-point bending experiment

The three-point bending experimental data needed in this study were obtained from our previous research, which focused on measuring the macro- and nano-levels mechanical parameters of the rat femoral cortical bone structures under treadmill running with different intensities [22]. In this experiment, five-month-old male Sprague–Dawley rats were regarded as the experimental objects. Forty-eight healthy rats were randomly assigned to the sedentary control group (SED, *n* = 12) and treadmill running groups with speeds of 12 m/min (EX12, *n* = 12), 16 m/min (EX16, *n* = 12), and 20 m/min (EX20, *n* = 12). Throughout the experimental period, the rats in the exercise groups ran on the treadmill for 30 min/day, 5 days a week, for 4 weeks. The rats in the sedentary control group were allowed to move freely in the cages. All the rats were killed, and their right femurs were harvested after one month of rearing.

The three-point bending experiment was performed on the femur samples to obtain the fracture load. The experimental span was set to 20 mm, and the indenter was loaded at a uniform speed of 0.5 mm/min downward. The bending load from the indenter was applied on the cortical bone structure in the middle of the femoral shaft. The nanoindentation test was performed on the cortical bone to measure the tissue-level elastic modulus. Two cortical bone structures with equal size were cut along the axis of the femoral shaft in each sample. One was used to measure the transverse elastic modulus, and the other measured the longitudinal elastic modulus. Nanoindentation tests were performed with a speed of 750 μN/s and an indentation depth of 1000 nm [22].

### Micro‑CT scanning

Forty-eight femurs were scanned by a micro-CT system at 18 μm voxel image resolution with 70 kV, and 100 μA. The scanned data were reconstructed using the NRecon software. The region of interest (ROI) was manually selected for the analysis of the femoral micro-CT images with the CTAn software. The total 5 mm cortical bone structure from the middle of the femoral shaft to the proximal and distal ends was selected as the ROI, where the failure location in the femur under three-point bending load was also in the range of this region. The microstructural parameters in the ROI of the femoral shaft cortical bone structure, including the cortical bone mineral density (Ct.BMD), cortical bone thickness (Ct.Th), and cortical bone porosity (Ct.P), were measured.

### Establishment of finite element models

**Fig. 4:**
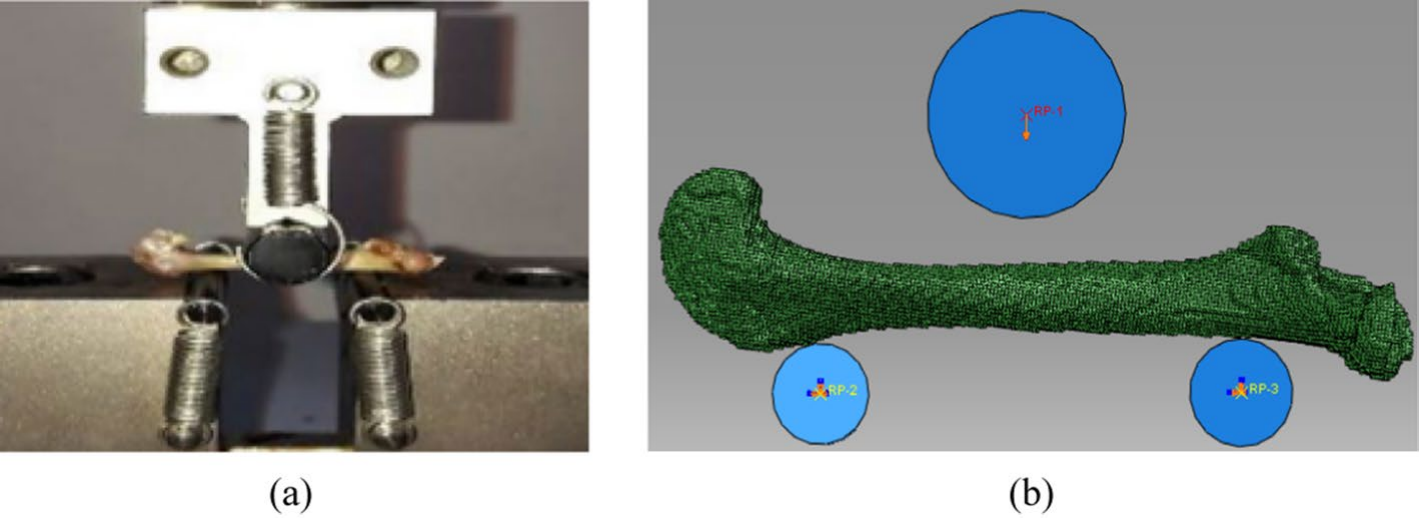
The schematic diagrams of the boundary condition on the rat femur under three-point bending. **a** the experimental boundary condition; **b** the simulated boundary condition

The femoral micro images were imported into the MIMICS software to reconstruct the geometric model. Because the failure region was located at the cortical bone structure in the middle of the femoral shaft during three-point bending load, the trabecular bone structure was not created in the FE model. The image segmentation was chosen in the range of 800–2000 HU, and the trabecular bone region in the MIMICS software was not selected during the geometric reconstruction process [34]. Then, the femur FE model was established by ABAQUS software using the C3D8 element. The upper rigid indenter and lower rigid cylinders were created above and below the femur FE model to simulate the experiment boundary condition. Due to the large surface roughness of the lower cylinders in the electronic testing machine, great friction can be provided between the lower surfaces of the cylinders and the femur. Therefore, the lower cylinders were fixed to the lower surface of the FE model using the TIE connection. The upper indenter was set to frictionless contact with the femur model. The upper indenter was compressed vertically downward, and the two lower cylinders were fully constrained to implement bending load. The loading schematic diagrams in the previous experiment and the present simulation can be seen in Fig. 4.

### Fracture simulation with finite element models

As observed in the previous experiment and literature, the quasi-brittle fracture occurred on the rat femoral cortical bone structure under three-point bending load [12, 22]. Therefore, the damage process can be described as the degradation of the structural mechanical properties when the critical failure strain in the element was reached [30]. This was accomplished using the ABAQUS User Material (UMAT) subroutine, where the material degradation was introduced to describe the progressive loss of stiffness due to crack initiation and propagation. In a quasi-static regime, the constitutive equation of elasticity introduced by a damage mechanics degradation can be expressed as [31, 32]:

1 $${\varvec{\upsigma}}={\mathbf{C}}_{\mathrm{d}} {\varvec{\upvarepsilon}}$$

2 $${\mathbf{C}}_{\mathrm{d}}=(1-\mathrm{D})\mathbf{C}$$

where **σ** is the element stress tensor, ${\mathbf{C}}_{\mathrm{d}}$ is the damaged stiffness matrix tensor in the element, **ε** is the element strain tensor, D is the damage variable, **C** is the initial stiffness matrix tensor in the element.

A key point in this fracture simulation is the choice of the damage evolution law describing the damage accumulation in the FE model. Because the bone failure process is mainly controlled by the element strain in the FE model, the damage evolution law was selected as [33]:

3 $$\mathrm{D}=0\mathrm{ for }(\upvarepsilon \le {\upvarepsilon }_{\mathrm{f}});\mathrm{ D}=1-{\mathrm{e}}^{\left(1-\frac{\upvarepsilon }{{\upvarepsilon }_{\mathrm{f}}}\right)}\mathrm{ for }(\upvarepsilon >{\upvarepsilon }_{\mathrm{f}})$$

where ε is the maximum or minimum principal strain in the element, ε_f_ is the critical tensile or compressive failure strain in the cortical bone material.

The structure remained in the elastic stage, and the initial stiffness matrix in the element was not degraded when the principal strain was less than the critical failure strain of the cortical bone material. The principal strain gradually exceeded the critical failure strain as the bending increased, and the structure entered into the damage stage. The damage variable *D* increased with the principal strain, causing a decrease in the element stiffness. The damaged stiffness matrix was adopted to update the stress and Jacobian matrix in the element. The Jacobian matrix after the onset of the damage could be described as expression (4) [34]. When the damage variable *D* approached 0.999, the element failed and lost its bearing capacity. With further bending, the cortical bone structure cannot be effectively loaded and underwent complete fracture when the failed element increased and accumulated to a certain degree.

4 $$\frac{\partial {\varvec{\upsigma}}}{\partial {\varvec{\upvarepsilon}}}={\mathbf{C}}_{\mathrm{d}}+\frac{\partial {\mathbf{C}}_{\mathrm{d}}}{\partial {\varvec{\upvarepsilon}}}: {\varvec{\upvarepsilon}}={\mathbf{C}}_{\mathrm{d}}+\frac{\partial {\mathbf{C}}_{\mathrm{d}}}{\partial \mathrm{D}}\times \frac{\partial \mathrm{D}}{\partial\upvarepsilon }$$

### Prediction process of micro-level failure strain

In the simulation process, the maximum and minimum principal strains in the element were compared with the critical tensile or compressive failure strains of the cortical bone material to determine whether damage occurred. Therefore, cortical bone fracture can be simulated by inputting four material parameters in the UMAT subroutine, namely, the elastic modulus, Poisson’s ratio, and critical tensile and compressive failure strains. First, the longitudinal and transverse tissue-level elastic moduli of the corresponding rat femoral cortical bone samples have been measured by previous nanoindentation test, as shown in Table 4 [22]. The poisson's ratio was set to 0.3 [1, 14]. Additionally, the ratio of critical tensile to compressive failure strain of the cortical bone material was set to 0.6 according to the literature [35, 36]. Therefore, only one material input parameter, namely the critical tensile or compressive failure strain, was not known. The critical tensile or compressive failure strain in the cortical bone material was the micro-level failure strain in the structure, which was the predicted object in the paper.

**Fig. 5:**
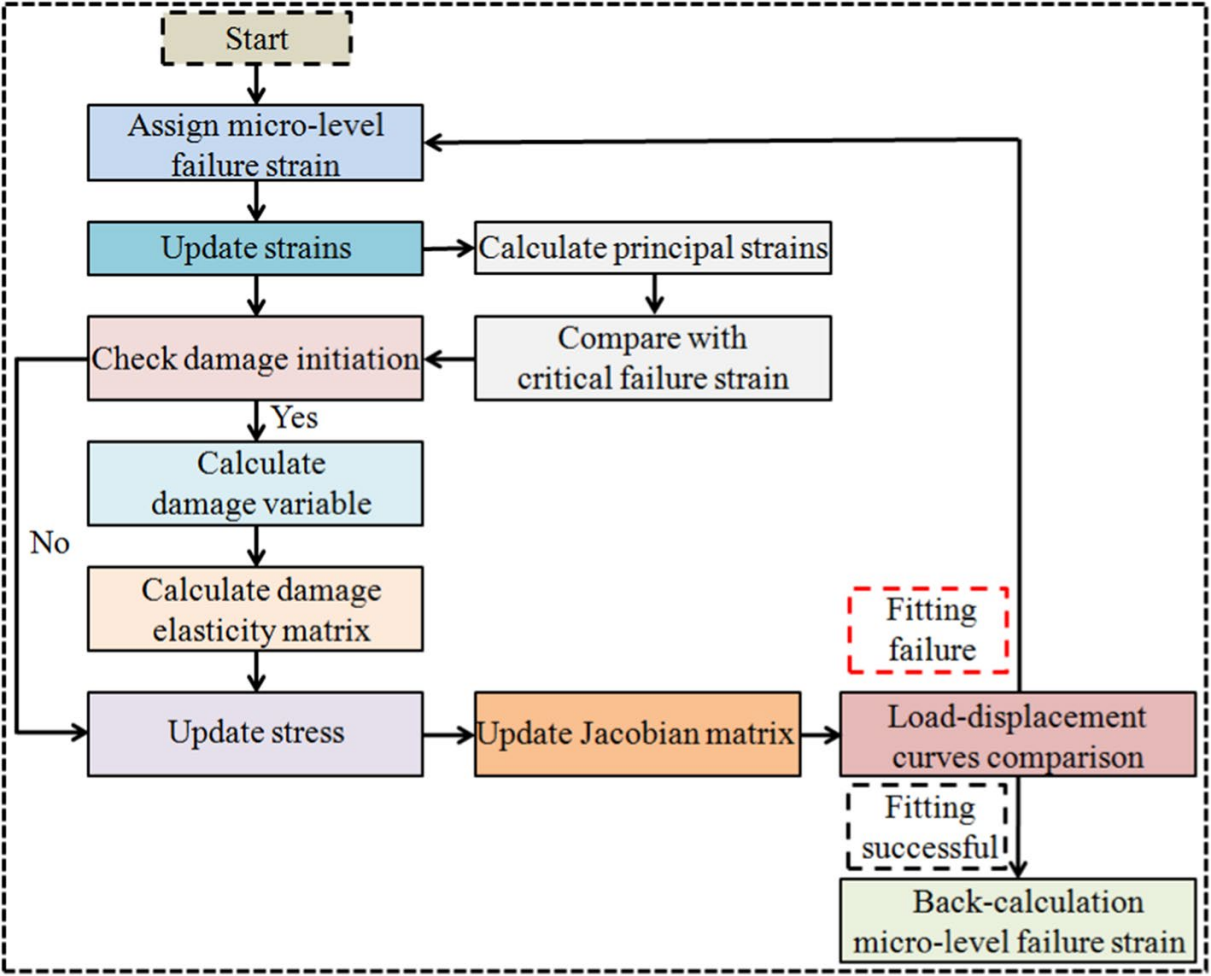
Flow chart of the prediction process for the micro-level failure strain

The first step was to select the approximate range of micro-level failure strain for the rat cortical bone structure. According to the literature, the failure strain of the cortical bone material is in the range of 0.01–0.05 [37, 38]. Thus, the prediction started with tensile and compressive failure strains of 0.01 and 0.0167, respectively. Every FE model was solved with a range of failure strains in tension and compression while maintaining 0.6 asymmetry until a single set of values could be determined that could make the simulated load–displacement curve best fit the experimentally measured one. Thus, the micro-level failure strain could be predicted by back-calculated fitting from the experimental results. The flowchart of the prediction process can be seen in Fig. 5.

### Mesh sensitivity analysis

**Fig. 6:**
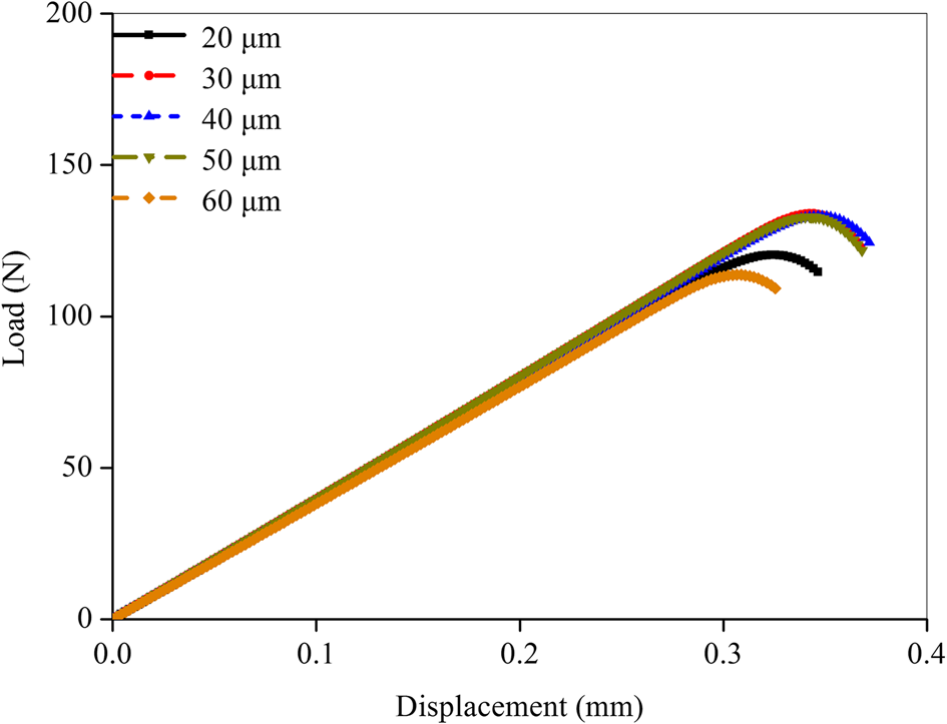
Mesh sensitivity analysis for the finite element models with different mesh size

Mesh sensitivity analysis was performed to determine the suitable element size for the femur FE model. Based on the femoral micro images from the SED group, different element sizes (20, 30, 40, 50, and 60 μm) were selected to establish the femur FE models. The load–displacement curves predicted in the simulations with the five FE models are shown in Fig. 6. The shape of the load–displacement curve was similar when the material input parameters were the same and only the element size was different. However, certain differences existed in the fracture load and fracture time. The FE model with coarse element underwent complete fracture early because the damage variable rose faster for the large element size, resulting in a faster decrease in the structural stiffness. The predicted three curves had no obvious differences in the fracture parameters when the element size was in the range of 30–50 μm. Therefore, the element size of the forty-eight femoral FE models established in this study was set at 30 μm considering the prediction accuracy.

### Statistical analysis

Data analysis was performed using SPSS software. The mean values of all the parameters in each group were calculated. Differences among all groups were analyzed by one-way analysis of variance. If significant differences were observed, the LSD post hoc test was used to compare the differences between every two groups. The significance level of *p* was chosen to be 0.05.

## Data Availability

The datasets used during the current study are available from the corresponding author on reasonable request.

## Abbreviation

FE: Finite element
